# *GlMPC* activated by GCN4 regulates secondary metabolism under nitrogen limitation conditions in *Ganoderma lucidum*

**DOI:** 10.1128/mbio.01356-23

**Published:** 2023-09-21

**Authors:** Zi Wang, Juhong Chen, Juan Ding, Jing Han, Liang Shi

**Affiliations:** 1 Key Laboratory of Agricultural Environmental Microbiology, Ministry of Agriculture Microbiology Department, College of Life Sciences, Nanjing Agricultural University, Nanjing, Jiangsu, China; Friedrich-Schiller-Universitat, Jena, Germany

**Keywords:** GCN4, GlMPC, nitrogen utilization, TCA, secondary metabolism

## Abstract

Fungi actively alter their metabolic and synthetic pathways to cope with changing environmental conditions. The mitochondrial pyruvate carrier (MPC) plays a vital role in transporting pyruvate into mitochondria and regulating carbon metabolic homeostasis. However, the mechanism by which MPC regulates carbon metabolism in response to environmental stress in fungi remains unclear. In this study, yeast one-hybrid library screening assays were employed to screen for transcription factors in *Ganoderma lucidum* that may interact with GlMPC. General control non-derepressible 4 (GCN4), a key transcription factor responding to nitrogen metabolism, was found to interact with the *GlMPC* promoter region. This interaction was further confirmed by electrophoretic mobility shift assays and chromatin immunoprecipitation quantitative PCR assays, demonstrating that GCN4 is capable of binding to the *GlMPC* promoter region *in vitro* and *in vivo*. Further results indicated that GCN4 could activate *GlMPC* expression and participate in regulating the tricarboxylic acid (TCA) cycle and ganoderic acid (GA) biosynthesis under low nitrogen conditions in *G. lucidum*. These findings revealed a specific regulatory mechanism by which GCN4-activated *GlMPC* regulates the TCA cycle and secondary metabolism under nitrogen limitation conditions in *G. lucidum*, providing crucial insights into the regulation of carbon and nitrogen metabolism in fungi.

**IMPORTANCE:**

Mitochondrial pyruvate carrier (MPC) is a pyruvate transporter that plays a crucial role in regulating the carbon metabolic flow and is considered an essential mechanism for microorganisms to adapt to environmental changes. However, it remains unclear how MPC responds to environmental stress in organisms. General control non-derepressible 4 (GCN4), a key regulator of nitrogen metabolism, plays a pivotal role in the growth and development of fungi. In this study, we report that GCN4 can directly bind to the promoter region and activate the expression of GlMPC, thereby regulating the tricarboxylic acid cycle and secondary metabolism under nitrogen limitation conditions in *Ganoderma lucidum*. These findings provide significant insights into the regulation of carbon and nitrogen metabolism in fungi, highlighting the critical role of GCN4 in coordinating metabolic adaptation to environmental stresses.

## INTRODUCTION

Changes in the growing environment, such as nutrient availability and extreme temperatures, could significantly affect the growth and development of organisms. Metabolic reprogramming is a fundamental activity continuously carried out by living organisms, helping them adapt to changing internal and external environments for optimal growth and survival (1). In particular, plants and microorganisms have limited life ranges and are difficult to migrate to more suitable environments. Thus, they must produce various metabolites to cope with diverse environments (2, 3). Recent studies show that metabolic rearrangements not only alter metabolite content but also modify intracellular environments, such as oxidation-reduction reactions (4). Therefore, studying metabolic regulation enables clearer analysis of microbial responses and adaptations to the environment (5). However, current studies on metabolic rearrangements mainly concentrate on carbohydrate metabolism, lipid metabolism, and amino acid biosynthesis (6 – 9). Less research has been conducted on key protein expression regulation in metabolic processes, especially mutual regulation between different metabolic pathways.

In organisms, mitochondria are organelles that perform core metabolic functions. Pyruvate is situated at the intersection of glycolysis, gluconeogenesis, and the tricarboxylic acid (TCA) cycle, and its transport into the mitochondrial matrix affects carbohydrate, fatty acid, and amino acid metabolism. Therefore, pyruvate metabolism has a significant impact on overall cell metabolism. The mitochondrial pyruvate carrier (MPC) is an essential transporter of pyruvate into mitochondria (10). MPC could regulate intracellular energy production by modifying the direction of carbon source metabolic flow, thereby allowing adaptation to changes in nutritional environments. In *Aspergillus oryzae*, a filamentous fungus, the loss of *MPC* restricts the transfer of pyruvate to mitochondria, leading to a metabolic shift from respiration for energy to efficient production of lactic acid or 2,3-butanediol, even under aerobic conditions (11). In *Saccharomyces cerevisiae*, MPC functions by regulating the rate of mitochondrial pyruvate transport, thus allowing cells to adapt to varying nutritional conditions (12). Furthermore, the expression level of *MPC* is typically downregulated in cancer cells, and they rely on glycolysis rather than oxidative phosphorylation for energy. Pyruvate is not transported to mitochondria, and cell growth is supported by glutamine oxidation to maintain the TCA cycle (13 – 15). MPC has also been found to be involved in abiotic stress resistance. In *Arabidopsis thaliana*, MPC plays a role in cadmium stress response (16). In addition, in *A. thaliana*, the negative regulator 1 (NRGA1) of guard cell abscisic acid (ABA) signaling encodes mitochondrial pyruvate carrier protein. Mutants with NRGA1 dysfunction have increased sensitivity to ABA and improved tolerance to drought stress (17). These findings indicated that MPC plays a crucial role in regulating the flow of carbon metabolism.

Recent years have seen an increase in research on the influence of environmental variables on *MPC* expression. In *Ganoderma lucidum*, silencing *GlMPC* reduced mycelium growth rates by 14–54% compared to wild-type (WT) strains when glucose, sucrose, or soluble starch was the only carbon source. These results demonstrated that the change in exogenous carbon sources can significantly affect the transcription level of *MPC* and consequently impact mycelia growth (18). Further studies on transcriptional regulation have demonstrated that nutrient- and environmental-related transcription factors regulate *MPC* transcription. In cholangiocarcinoma cells, peroxisome proliferator-activated receptor γ coactivator-1α (PGC1α) upregulates the expression of *MPC1* to transport pyruvate into mitochondria for oxidation and mediate metabolic switch to oxidative phosphorylation (19). In *S. cerevisiae*, the core transcription factor Sko1 in the hyperosmotic glycerol (HOG) mitogen-activated protein kinase pathway could directly bind to the promoter of *MPC3* and mediate transcriptional response to osmotic stress (20). These findings highlight the importance of transcriptional regulation in controlling metabolic flux and energy usage in metabolic processes through MPC.

*G. lucidum* is a basidiomycetes species widely cultivated in Asia that produces various secondary metabolites with significant bioactivity. With the completion of genome sequencing and the development of transgenic systems, *G. lucidum* has become a model organism for investigating secondary metabolic regulation in macrofungi. Some studies show that *G. lucidum* could regulate metabolic flow and increase the accumulation of ganoderic acids (GA), which are key secondary metabolites, in response to changes in environmental signals (21 – 27). Salicylic acid inhibits complex III activity, which generates reactive oxygen species, and thereby induces GA overproduction in *G. lucidum* (21). Under heat stress, the activity of Glsnf1 increases. The activation of Glsnf1 participates in the metabolic shift from respiration to glycolysis, which helps *G. lucidum* cope with heat stress-induced ROS damage by activating the ROS scavenging system and GA biosynthesis (5). In *G. lucidum*, two homologous *MPC* genes (*GlMPC1* and *GlMPC2*) have been identified and are responsive to changes in environmental nutrition, influencing carbon metabolism in *G. lucidum* (18). Studies have demonstrated that silencing *GlMPC* can enhance the utilization of non-dominant carbon sources by increasing the intracellular glycolysis rate, the consumption rate of carbon sources, and reducing glucose accumulation (18). Knockdown of *GlMPC* reduces pyruvate content in mitochondria, thereby altering fatty acid metabolism and leading to GA accumulation. These findings suggest that GlMPC plays a significant role in metabolic rearrangement in *G. lucidum*. However, it remains unclear how MPC contributes to environmental effects on metabolic rearrangement in *G. lucidum*.

This study employed yeast one-hybrid (Y1H) library screening assay to screen transcription factors that potentially interact with MPC in *G. lucidum*. General control non-derepressible 4 (GCN4), a key transcription factor regulating core nitrogen metabolism, was found among these potential key transcription factors. It was found that GCN4 could directly bind to *GlMPC* promoter regions, activate *GlMPC* expression, and participate in the regulation of secondary metabolism under low nitrogen conditions. This research revealed the regulatory mechanism for nitrogen utilization in *G. lucidum* through GCN4 and MPC, providing a valuable strategy for regulating nitrogen utilization in *G. lucidum*.

## RESULTS

### GCN4 directly binds and activates the expression of *GlMPC1/2*

MPC plays a crucial role in regulating the metabolism of mitochondrial carbon sources. To explore the regulatory mode of GlMPC at the transcriptional level, the Y1H screening assay was performed using the *G. lucidum* cDNA library. The 1,000 bp fragment of the *GlMPC1* promoter was cloned into pAbAi to generate the reporter vector. A total of 132 clones were screened, with 15 positive clones independently interacting with the *GlMPC* promoter region (Table S1). Among the transcription factors identified were three types: FUNgal_trans, bHLHzip_Myc, and bZIP_GCN4, which have multiple functions, including sensing changes in the external environment and regulating intracellular physiological metabolism (28 – 30). These results suggested that GlMPC is regulated by multiple transcription factors and participates in many environmental responses. Notably, among these transcription factors was GCN4 (basic leucine zipper), which has been widely reported to interact with the UAS_GCRE_ (GA [C/G] TCA) motif in the promoter region (28, 30).

**Fig 1:**
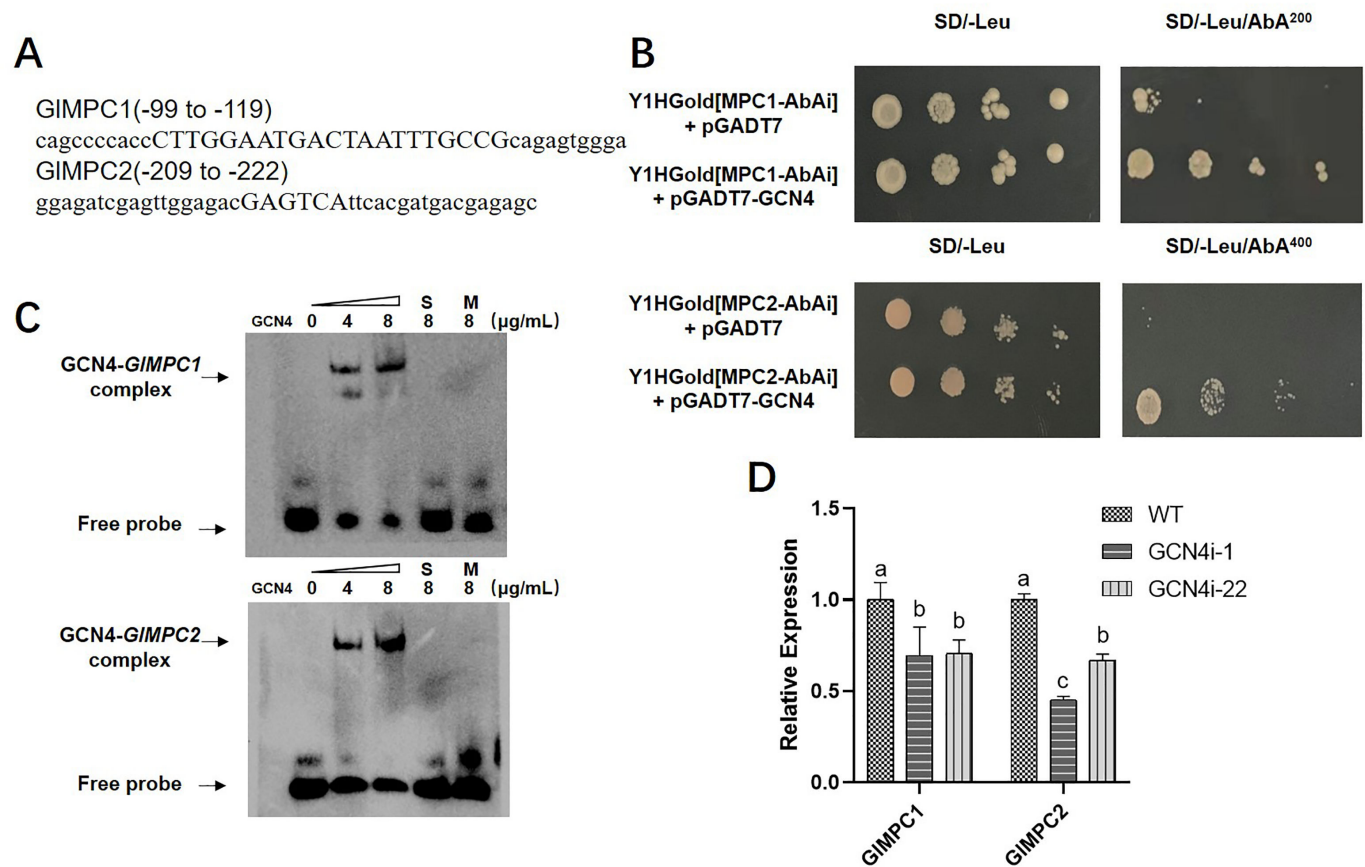
GCN4 directly binds and activates the expression of *GlMPC1/2*. (A) Schematic diagram of the consensus GCN4-binding sites in the promoter regions of *GlMPC1/2*. The predicted binding site in promoter regions of *GlMPC1* was 21 bp. The UAS_GCRE_ in promoter regions of *GlMPC2* was 6 bp. (B) The binding of GCN4 to the promoter region of *GlMPC1/2* genes with the Y1H assay. The vector contained GCN4 in the Y1HGold yeast strain mixed with the vector contained promoter region of *GlMPC1/2*, gradient dilution, and dotted in the plates of SD/-Leu, SD/-Leu/AbA^200^, and SD/-Leu/AbA^400^. The growth of yeast was detected by static culture at 30℃ for 3 days. (C) Electrophoretic mobility shift assay verified the binding effect of GCN4 to the promoter region of *GlMPC1/2* genes. The amounts of purified recombined GCN4 used were approximately 0–8 μg·mL^−1^, and about 10 ng of the biotin-labeled probe (promoter fragments of *GlMPC1/2*) was added to each reaction. The 100-fold dilution of the unlabeled specific probe (S) was used as competitor DNA. A sequence mutation-binding probe (M) was used as a mutational probe to test for specificity. (D) The expression levels of *GlMPC1/2* in *GCN4*-silenced strains were detected by RT-qPCR. Data are presented as the mean ± SD (*n* = 3). Statistical significance is represented by different letters corresponding to *P* < 0.05 based on Tukey’s multiple range test.

To explore whether GCN4 binds to the promoter region of *GlMPC1* and *GlMPC2*, *cis*-acting element prediction was conducted on the promoter of *GlMPC1* and *GlMPC2*. The prediction results revealed a highly conserved GCN4 recognition element, the UAS_GCRE_ binding element, at −119 to −99 bp in the *GlMPC1* promoter and at −222 to −209 bp in the *GlMPC2* promoter (Fig. 1A). Moreover, Y1H was performed, and the results (Fig. 1B) demonstrated that GCN4 bound to the predicted promoter fragment containing the UAS_GCRE_ motif in the *GlMPC1/2* promoter. Thus, these findings suggested that GCN4 is capable of binding to the promoter of *GlMPC1/2*.

To further confirm the binding specificity of GCN4 to the recognition element in *GlMPC1/2*, an electrophoretic mobility shift assay (EMSA) was performed utilizing a GlGCN4-pCold I fusion protein. The fragments from the *GlMPC1/2* promoter region containing the predicted GCN4 recognition element with a length of 41 bp were labeled with 5′-biotin and used as probes in EMSA (Table S2). Incubation of the GlGCN4 protein/*GlMPC1/2* probe complex with increasing concentrations of GlGCN4 protein led to a dose-dependent increase in the shifted band (Fig. 1C). In competitive binding experiments, 100-fold excesses of unlabeled DNA probes were included in the binding reaction. It was found that no binding occurred when the binding motif in these genes was mutated (Fig. 1C), indicating that the binding of GCN4 to the motif in the *GlMPC1/2* promoter was specific. Therefore, Y1H and EMSA results confirmed that GCN4 directly binds to the predicted binding motifs in the promoter of *GlMPC1/2*.

Moreover, the expression of *GlMPC* was detected in *GCN4*-silenced strains previously constructed (3). The results showed that in *GCN4*-silenced strains, the expression of *GlMPC1* was downregulated by approximately 30%, and that of *GlMPC2* was downregulated by 33–55%, compared to the WT (Fig. 1D). These findings indicated that GCN4 could directly bind to the promoter region of *GlMPC1/2* and activate the expression of *GlMPC1/2*.

### GlMPC is involved in the metabolism regulation of nitrogen limitation in *G. lucidum*

**Fig 2:**
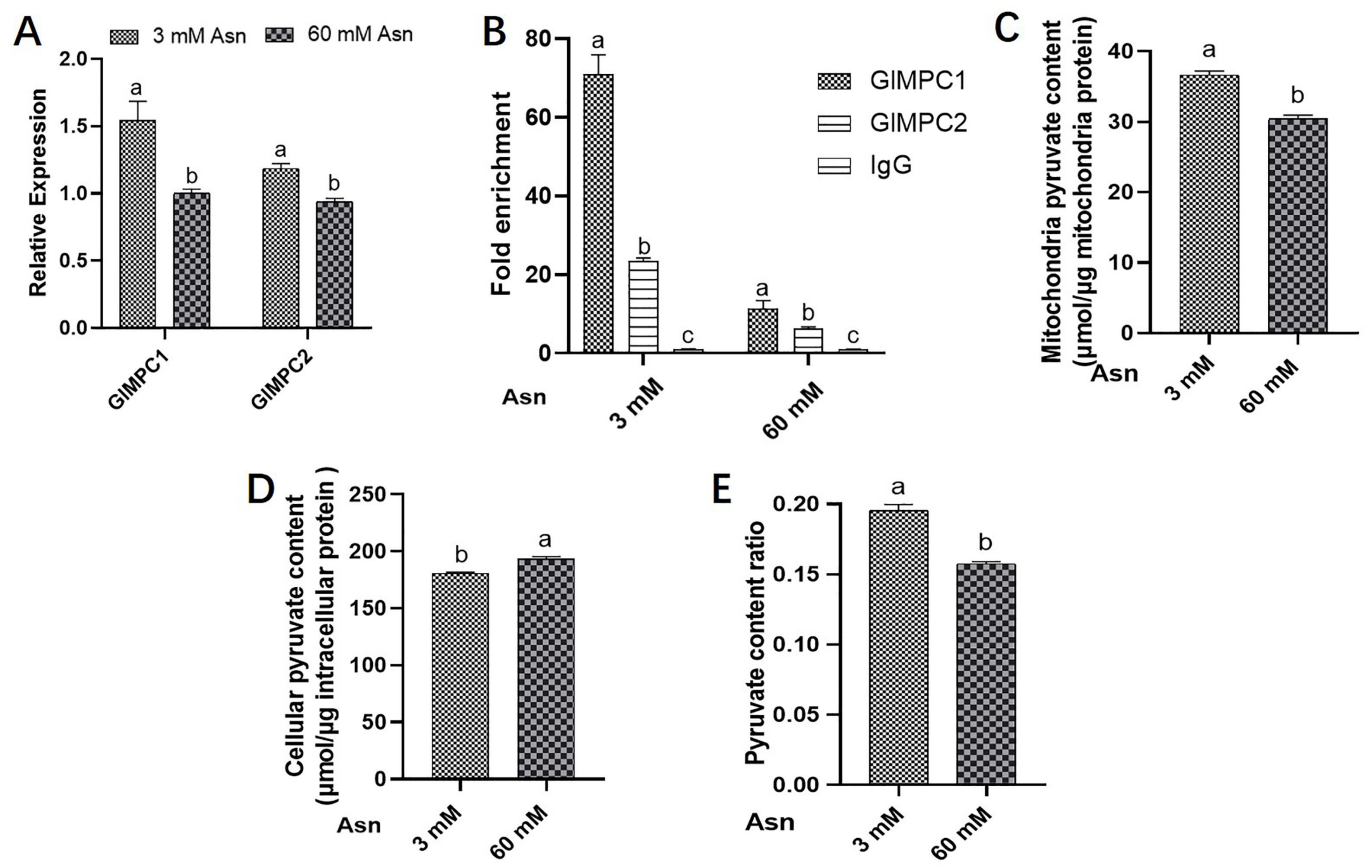
GlMPC activity responds to nitrogen limitation conditions in *G. lucidum*. (A) The expression levels of *GlMPC1/2* under 3 mM and 60 mM Asn were detected by RT-qPCR. (B) ChIP-qPCR assays were performed on 1-week-old hyphae under 3 mM and 60 mM Asn to show the relative binding strength of GCN4 to the *GlMPC1/2* promoter. Immunoprecipitate (IP)/input was calculated by comparison with the threshold cycle (C_T_) values between the immunoprecipitate and input. (C) Mitochondria pyruvate content under 3 mM and 60 mM Asn. (D) Cellular pyruvate content under 3 mM and 60 mM Asn. (E) The ratio of mitochondrial pyruvate content to intracellular pyruvate content under 3 mM and 60 mM Asn. Data are presented as the mean ± SD (*n* = 3). Statistical significance is represented by different letters corresponding to *P* < 0.05 based on Tukey’s multiple range test.

Previous studies have established that GCN4 is an essential transcription factor in nitrogen metabolism and has garnered extensive attention (3, 28). To investigate whether GlMPC responds to nitrogen limitation stress in *G. lucidum*, the expression levels of *GlMPC1*/*2* were detected through RT-qPCR with 60 mM or 3 mM asparagine (Asn) as the sole nitrogen source. As shown in Fig. 2A, compared with 60 mM Asn treatment, the expression levels of *GlMPC1/2* were increased by 50% and 20% in the 3 mM Asn treatment group, respectively (Fig. 2A). These results suggested that nitrogen limitation conditions could active *GlMPC* expression in *G. lucidum*. To explore the difference in the ability of GCN4 to bind the *GlMPC1/2* promoter under nutrient-rich and nitrogen-limited conditions, chromatin immunoprecipitation quantitative PCR (ChIP-qPCR) assays were performed using *GlMPC1/2* targets incubated with anti-GCN4 antibody (Fig. 2B). The results revealed that the GCN4 immunoprecipitated genomic DNA under 3 mM Asn conditions exhibited 6.5- and 3.5-fold higher enrichment in the *GlMPC1* and *GlMPC2* promoter regions, respectively, compared to the samples treated with 60 mM Asn. Moreover, under the condition of 60 mM Asn, the GCN4 immunoprecipitated genomic DNA exhibited a 1.8-fold higher abundance in the *GlMPC1* promoter region (spanning from −119 bp to −99 bp) compared to the *GlMPC2* promoter region (spanning from −222 bp to −209 bp). However, at 3 mM Asn, the GCN4 immunoprecipitated genomic DNA showed a 3.1-fold higher enrichment in the *GlMPC1* promoter region than in the *GlMPC2* promoter region. These findings suggested that GCN4 has a higher affinity for the promoter region of *GlMPC1* compared to *GlMPC2*, both under nitrogen-limited and nitrogen-rich conditions. Furthermore, GCN4 demonstrates stronger binding to the *GlMPC1/2* promoter region under nitrogen-limited conditions than under nitrogen-rich conditions.

To further study the impact of nitrogen limitation conditions on the function of GlMPC in *G. lucidum*, the ratio of mitochondrial and intracellular pyruvate content under nitrogen limitation treatment was used to represent the level of mitochondrial pyruvate transport rate. As shown in Fig. 2C and D, compared to the pyruvate content under the 60 mM Asn treatment, the pyruvate content in the mitochondria in the 3 mM Asn treatment group increased by 6%, while the total pyruvate content decreased by about 20%. In addition, it was found that the ratio of mitochondrial pyruvate content to total pyruvate content was significantly increased by about 27% under nitrogen limitation conditions (Fig. 2E). These findings suggested that the activity of *GlMPC1/2* is significantly upregulated in response to nitrogen limitation treatment, and nitrogen metabolism has a significant impact on mitochondrial pyruvate transport in *G. lucidum*.

**Fig 3:**
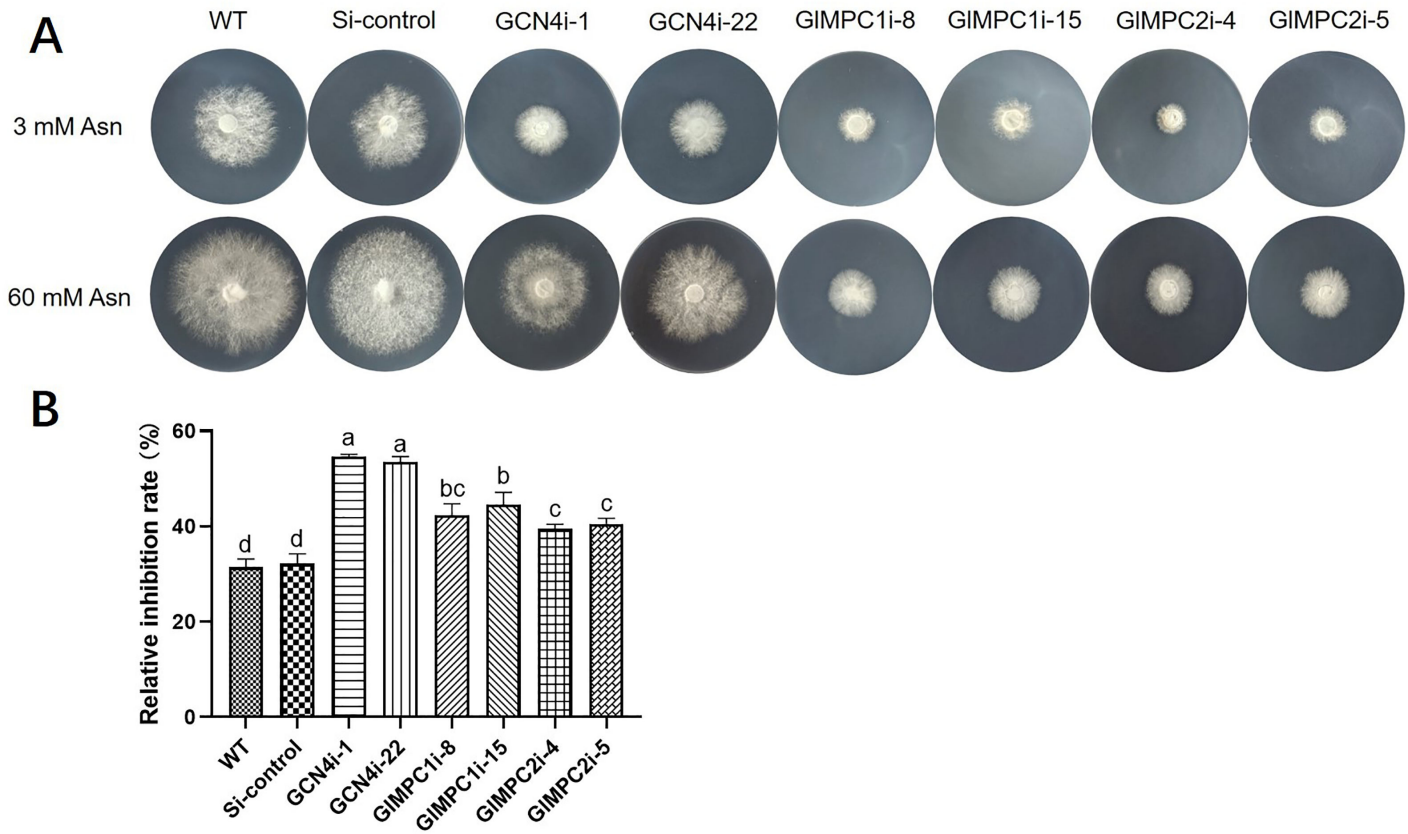
Mitochondrial pyruvate transport was significantly increased under nitrogen limitation conditions. (A) Images of mycelial growth in WT, Si-control, *GCN4*-silenced, and *GlMPC1/2*-silenced strains under 3 mM and 60 mM Asn. (B) Relative inhibition rates under 3 mM and 60 mM Asn in WT, Si-control, *GCN4*-silenced, and *GlMPC1/2*-silenced strains. The growth inhibition rate in each strain was calculated as follows: [diameter (60 mM Asn) − diameter (3 mM Asn)]/diameter (60 mM Asn)]. Data are presented as the mean ± SD (*n* = 3). Statistical significance is represented by different letters corresponding to *P* < 0.05 based on Tukey’s multiple range test.

To gain further insight into the contribution of GlMPC to the nitrogen utilization process, the growth difference of WT, Si-control, *GCN4*-silenced, and *GlMPC*-silenced strains was investigated under different nitrogen concentration conditions. The mycelia of these strains were, respectively, cultured in solid media with 3 mM or 60 mM Asn to monitor the growth of mycelia (Fig. 3). The results indicated a significant growth inhibition for all strains under the 3 mM Asn condition compared to the 60 mM Asn condition. Specifically, the relative growth inhibition rate of the WT and Si-control strains was 32%. While the relative growth inhibition rates for the GCN4i-1 and GCN4i-22 strains were 54% and 53%, respectively. The relative inhibition rates for the GCN4i-1 and GCN4i-22 strains showed an increase of 22% and 21%, respectively, compared to the WT and Si-control strains. Furthermore, the relative growth inhibition rates of *GlMPC*-silenced strains fell between those of the WT and *GCN4*-silenced strains. Specifically, the GlMPC1i-8 and GlMPC1i-15 strains exhibited an increase of 10% and 12%, respectively, in relative growth inhibition compared to the WT and Si-control strains. Similarly, the GlMPC2i-4 and GlMPC2i-5 strains showed a relative growth inhibition increase of 7% and 8% compared to the WT and Si-control strains. These results indicated that nitrogen utilization is directly affected in *GlMPC*-silenced strains.

### GlMPC and GCN4 affect key enzyme activity and gene transcription in the TCA cycle

**Fig 4:**
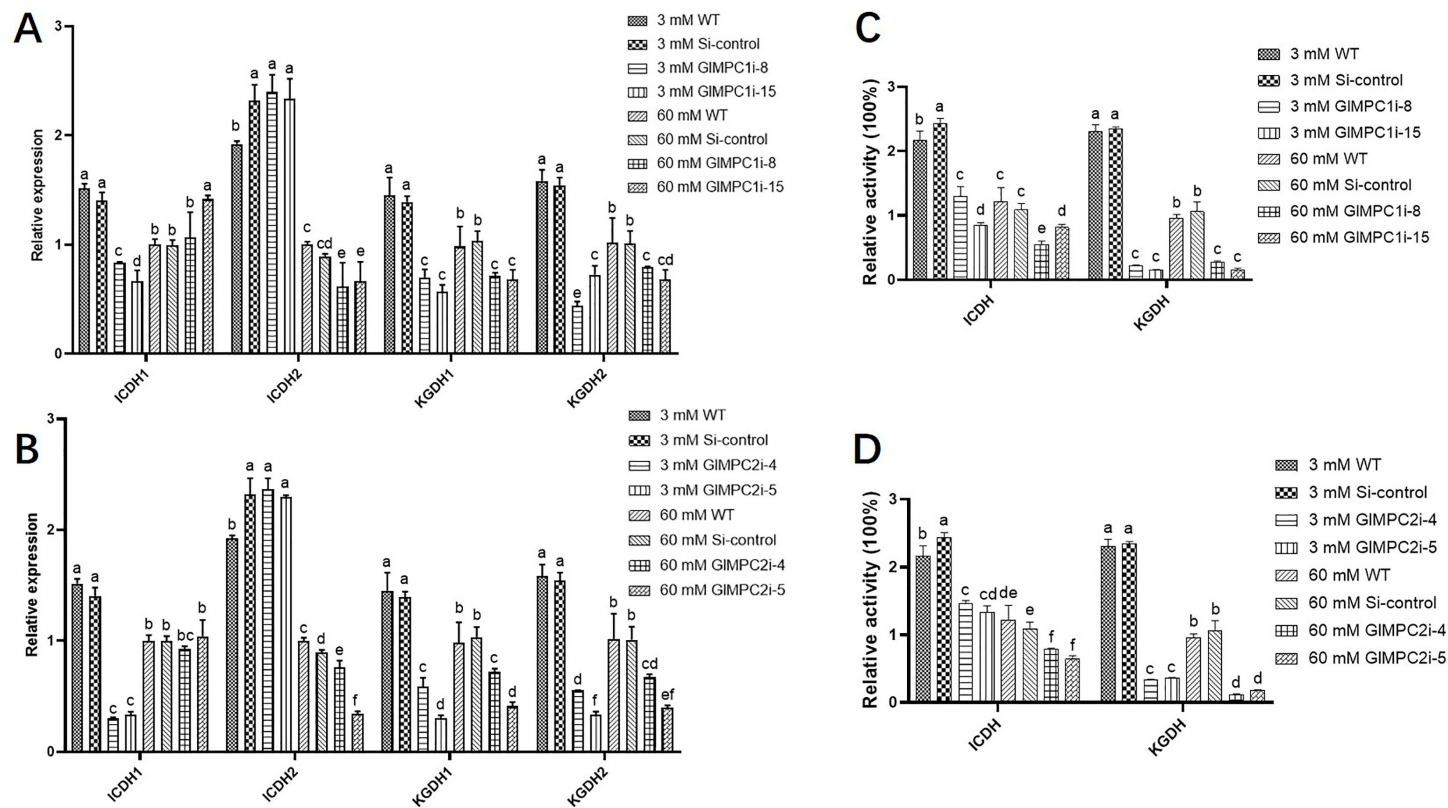
GlMPC is involved in regulating the TCA cycle under nitrogen limitation conditions. (A) Relative expression of genes related to key enzymes of the TCA cycle in WT, Si-control, and *GlMPC1*-silenced strains under 3 mM and 60 mM Asn. (B) Relative expression of genes related to key enzymes of the TCA cycle in WT, Si-control, and *GlMPC2*-silenced strains under 3 mM and 60 mM Asn. (C) The relative key enzyme activity of the TCA cycle, ICDH and KGDH, in WT, Si-control, and *GlMPC1*-silenced strains under 3 mM and 60 mM Asn. (D) The relative key enzyme activities of the TCA cycle, ICDH and KGDH, in WT, Si-control, and *GlMPC2*-silenced strains under 3 mM and 60 mM Asn. Data are presented as the mean ± SD (*n* = 3). Statistical significance is represented by different letters corresponding to *P* < 0.05 based on Tukey’s multiple range test.

Pyruvate is transported into the mitochondria, facilitated by the MPC, and is converted by pyruvate dehydrogenase (PDH) to acetyl CoA which enters the TCA cycle. To investigate the regulatory effects of GlMPC on the TCA cycle under low nitrogen conditions, the expression and activities of ICDH and KGDH, two key enzymes in the TCA cycle, in both WT and *GlMPC*-silenced transformants were detected. As shown in Fig. 4, compared with the 60 mM Asn treatment, the enzyme activities of two key enzymes and the expression of their corresponding synthetic genes were significantly upregulated in the 3 mM Asn treatment, indicating that a low concentration of nitrogen could promote the TCA cycle. Furthermore, the effect of silencing *GlMPC* on the TCA cycle under both 3 mM Asn and 60 mM Asn conditions was further detected. The results showed that silencing *GlMPC* significantly reduced the activities of key enzymes in the TCA cycle, compared to WT under 3 mM Asn conditions, with KGDH being most significantly reduced to 84.37–93.49% (Fig. 4C and D). However, under the condition of 60 mM Asn, silencing *GlMPC* also had a major effect on KGDH, which decreased to 78-84% (Fig. 4C and D). These findings suggested that GlMPC participated in positively regulating the TCA cycle under conditions of low nitrogen.

**Fig 5:**
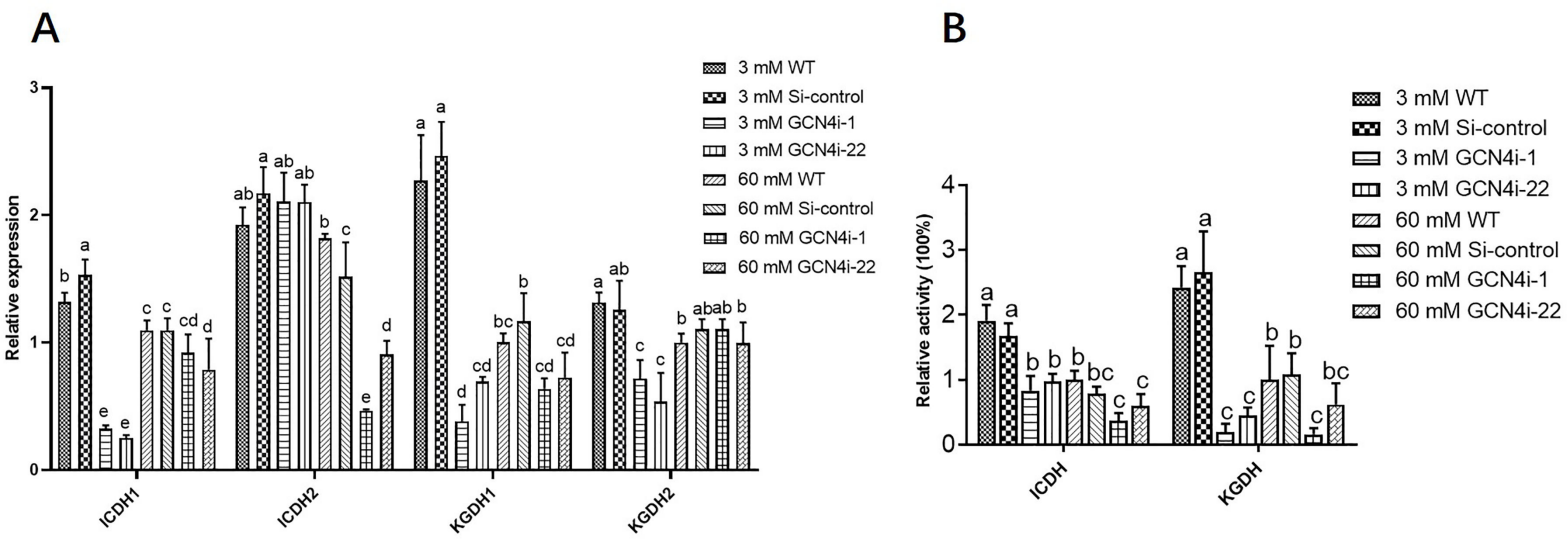
GCN4 is involved in regulating the TCA cycle under low nitrogen conditions. (A) Relative expression of genes related to key enzymes of the TCA cycle in WT, Si-control, and *GCN4*-silenced strains under 3 mM and 60 mM Asn. (B) The relative key enzyme activity of the TCA cycle, ICDH and KGDH, in WT, Si-control, and *GCN4*-silenced strains under 3 mM and 60 mM Asn. Data are presented as the mean ± SD (*n* = 3). Statistical significance is represented by different letters corresponding to *P* < 0.05 based on Tukey’s multiple range test.

It has been confirmed that GCN4 could bind to the promoter region of *GlMPC* and activate the expression of *GlMPC*. Therefore, the influence of GCN4 on the TCA cycle under low nitrogen was further investigated. The results showed that compared to 60 mM Asn treatment, activities and expression of ICDH and KGDH were significantly upregulated under 3 mM Asn condition (Fig. 5). The effect of silencing GCN4 in the TCA cycle under both 3 mM Asn and 60 mM Asn treatments was further detected. The results showed that under the condition of 3 mM Asn, the activity of ICDH and KGDH in the TCA cycle was significantly decreased in *GCN4*-silenced strains compared to the WT strain. The decrease in KGDH activity was most significant, resulting in an 86.8% decrease in activity (Fig. 5B). These findings indicated that GCN4 also positively regulates the TCA cycle under 3 mM Asn condition. Considering the results of GCN4 binding to the *GlMPC* promoter region, it is speculated that GCN4 could regulate TCA through GlMPC.

### GlMPC regulates GA biosynthesis under nitrogen limitation conditions

**Fig 6:**
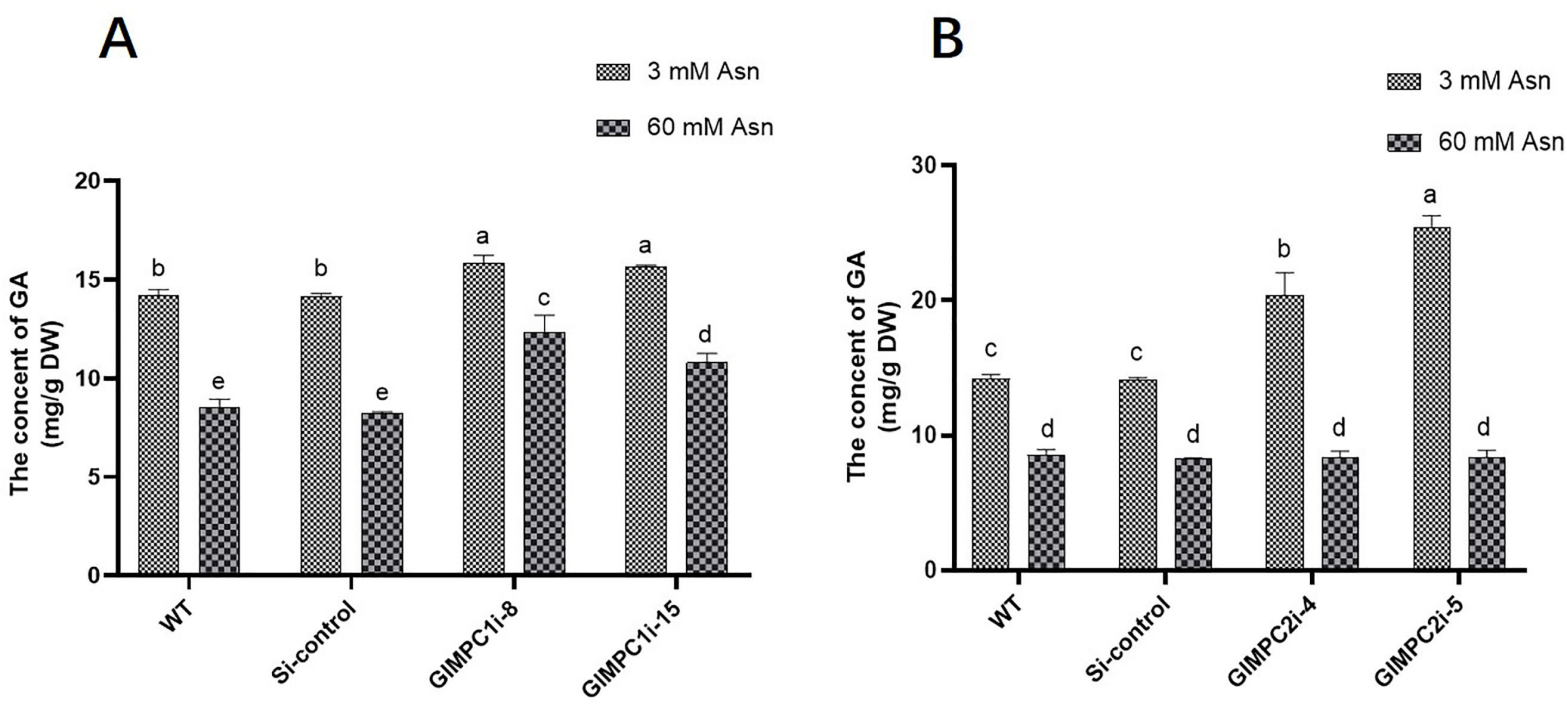
GlMPC regulates GA biosynthesis under nitrogen limitation conditions. (A) GA content in WT, Si-control, and *GlMPC1*-silenced strains under 3 mM and 60 mM Asn. (B) GA content in WT, Si-control, and *GlMPC2*-silenced strains under 3 mM and 60 mM Asn. Data are presented as the mean ± SD (*n* = 3). Statistical significance is represented by different letters corresponding to *P* < 0.05 based on Tukey’s multiple range test.

Previous studies have shown that nitrogen limitation can regulate GA biosynthesis through GCN4 (3). To explore whether GlMPC has involved in GA biosynthesis under nitrogen limitation, GA content in *GlMPC1/2*-silenced strains under 3 mM and 60 mM Asn conditions was measured. The results showed that, compared to mycelium treated with 60 mM Asn, the content of GA in mycelium treated with 3 mM Asn increased by 1.7-fold (Fig. 6), which was consistent with the previous results (3). Under 3 mM Asn treatment, silencing *GlMPC* significantly increased the GA content by 1.1-fold in *GlMPC1*-silenced strains (Fig. 6A), and by 1.4- and 1.8-fold in *GlMPC2*-silenced strains (Fig. 6B), compared with WT strains. However, there were no significant differences between *GlMPC*-silenced and WT strains under nitrogen abundance conditions. These findings indicated that silencing *GlMPC* could promote GA biosynthesis under nitrogen limitation.

## DISCUSSION

Environmental adaptation is crucial for organisms, and nutrient utilization is a key factor in this process (31 – 33). MPC plays a crucial role in the transition of carbon metabolic flow, regulating energy metabolism, cytoskeleton synthesis, secondary metabolism, and other crucial aspects; besides, it is considered an essential way for microorganisms to respond to environmental changes. For instance, upregulating GlMPC can lead to the transfer of pyruvate into the mitochondria, which enters into the TCA cycle, subsequently affecting primary metabolism (34, 35). Previous studies have demonstrated that MPC1 is essential for *A. thaliana* in maintaining the TCA cycle to support sustainable plant growth, indicating the importance of MPC in diverse organisms (36). GlMPC is also involved in regulating the accumulation of secondary metabolites caused by changes in carbon metabolic flow in *G. lucidum*, although the mechanism of how GlMPC responds to environmental signals remains unclear. In this paper, we discovered that GCN4 could bind to the promoter of *GlMPC* and initiate *GlMPC* transcription. Previous studies have indicated that nitrogen limitation can highly induce the expression of GCN4 and promote GA biosynthesis in *G. lucidum* (3). This study further demonstrated that GCN4 can directly bind and activate *GlMPC* expression, thereby affecting the ratio of pyruvate content inside and outside mitochondria, ultimately changing the carbon metabolism of *G. lucidum*. The findings in this study could enhance our understanding of the regulation of carbon metabolism in response to nitrogen sources.

MPC plays a crucial role in coordinating glycolysis and mitochondrial activity by serving as the only entry point for pyruvate into the mitochondrial matrix. As such, MPC can influence the TCA cycle by altering mitochondrial pyruvate content and plays a significant role in regulating metabolic flow. In cancer studies, the luciferase gene linked to the MPC promoter demonstrated that the chicken ovalbumin upstream promoter-transcription factor II (COUP-TFII) represses *MPC1* expression in prostate cancer cells, leading to a metabolic switch toward increased glycolysis and promotes cancer progression (37). Similarly, under starvation conditions, cAMP-responsive element-binding protein (CREB) was found to bind to the MPC promoter and regulate its transcription level to raise blood sugar levels (38). Moreover, lysine demethylase 5A can directly bind to the MPC-1 promoter region and inhibit *MPC-1* expression by demethylating H3K4 transcription (39). However, there are relatively few studies on the transcriptional regulation of *MPC*, particularly in microorganisms. In this study, the Y1H analysis revealed that GlMPC could potentially be regulated by FUNgal_trans, bHLHzip_Myc, and bZIP_GCN4 transcription factors. This suggested that the entry of pyruvate into the mitochondria is influenced by multiple factors and the need for further investigation into the specific molecular mechanism. Taken together, these findings contribute to our understanding of the role of MPC in environmental responses and their involvement in regulating intracellular metabolic flow.

Nitrogen is an essential component of the nucleic acids and proteins in organisms and is often a limiting factor in their metabolism, growth, and development. Research in mammals has found that the mammalian target of rapamycin complex 1 (mTORC1) promotes the utilization of glutamine by activating glutamate dehydrogenase (GDH), which then provides metabolites for the TCA cycle (40). In addition, studies in microorganisms have indicated that nitrogen limitation triggers morphological transitions in *S. cerevisiae* (41), *Aspergillus nidulans* (42), and *Neurospora crass* (43). In addition, nitrogen restriction has been found to significantly impact carbon metabolism and secondary metabolite accumulation in fungi. Research on microalgae has reported that nitrogen starvation is an effective strategy for inducing carbohydrate and lipid accumulation, as evidenced by multiple studies (3, 44, 45). Furthermore, proteomic analysis in *G. lucidum* has shown that, under nitrogen-restricted conditions, carbon skeletons integrated into GA precursors were regenerated by glycolysis and the TCA cycle to form amino acids, which act as a strategy to reserve nitrogen (46). Interestingly, it has also been found that low concentrations of nitrogen promote the synthesis of polysaccharides and crude proteins in *G. lucidum* (26, 47 – 49). However, studies on the regulation of nitrogen sources on carbon metabolism mainly focus on the changes in metabolic flow, and the mechanism by which nitrogen sources regulate carbon metabolism remains unclear. Nitrogen starvation may impact carbon metabolic flow and secondary metabolism through two regulatory pathways. The first pathway, nitrogen catabolite repression (NCR), is activated in response to the use of non-preferred nitrogen sources (50). The second pathway is general amino acid control (GAAC), which stimulates amino acid biosynthesis and nitrogen utilization through the critical transcription factor GCN4 (51, 52). GCN4 is considered a key regulator of core nitrogen metabolism, and it has recently been found to be involved in the regulation of secondary metabolism in fungi. For instance, in *Fusarium oxysporum*, cross-pathway control (CPC1), proteins homologous to GCN4, also interrupt the communication between the fungus and its host plants by affecting the production of secondary metabolites (53). In *G. lucidum*, it has been found that GCN4 influences the TCA cycle and GA biosynthesis under low nitrogen conditions (3). Therefore, studying the regulation mechanism of carbon metabolism by GCN4 is crucial to further our understanding of the regulation network of carbon and nitrogen sources in filamentous fungi.

As an essential nitrogen transcriptional regulator, GCN4 could activate more than 500 genes through its transcriptional regulatory function (28). Research indicates that GCN4 plays a transcriptional regulatory role by identifying and binding to the UAS_GCRE_ (GA [C/G] TCA) motif in the promoter region of target genes (52, 54). The majority of GCN4-regulated genes are related to nitrogen synthesis, along with autophagy-related proteins and intermediate metabolite-related synthesis genes of the TCA cycle and glycolysis pathway (28, 50, 52). In *G. lucidum,* GCN4 is also a critical transcription factor that regulates secondary metabolism. For example, it is reported that GCN4 affects GA biosynthesis by altering the metabolic flow of carbon sources, but the specific mechanism remains unclear (3).

As a key element of basic cell metabolism, MPC plays an important role in responding to abiotic stress. Several studies have reported that MPC1 functions as a protective mechanism against Cd stress in *Arabidopsis*. It accomplishes this by preserving the integrity of the TCA cycle and maintaining optimal ATP levels, while concurrently impeding the influx of Cd^2+^ (16). However, there is limited research on the underlying mechanism of MPC in response to nitrogen source utilization. Previous studies have shown that Asn, as the dominant nitrogen source, can regulate the biosynthesis of secondary metabolite in *G. lucidum* (3, 49). Therefore, to further analyze the molecular mechanism of *G. lucidum* response to nitrogen source regulation, 3 mM or 60 mM Asn was used as the only nitrogen source to simulate nitrogen limitation and nitrogen enrichment. This study conducted Y1H assays and EMSA to show that under low nitrogen conditions, GCN4 binds directly to the UAS_GCRE_ (GA [C/G] TCA) motif in the *GlMPC* promoter region of target genes, specifically activating *GlMPC* transcription (Fig. 1). Furthermore, Chip-qPCR demonstrated that GCN4 bound to the promoter region of *GlMPC1* more strongly than *GlMPC2* under nitrogen limitation. It indicated that GlMPC1 plays a more important function under nitrogen limitation. Therefore, in response to a low nitrogen environment, GCN4 activated *GlMPC* transcription, thereby affecting pyruvate entry into mitochondria and regulating the TCA cycle. The TCA cycle is not only critical for carbon source metabolism but also critical for amino acid metabolism (55). The synthesis and transportation of intracellular amino acids are essential processes in amino acid metabolism. Many intermediate metabolites produced during the TCA cycle also contain precursors for amino acid synthesis, serving as supplementary pathways to promote intracellular amino acid synthesis (56, 57). This study found that GCN4 could directly regulate the expression of *GlMPC*, further enhancing our understanding of the role of GCN4 in regulating carbon sources. Silencing GCN4 would reduce the transcription of *GlMPC*, increasing mitochondrial pyruvate content and altering the rate of the TCA cycle. All in all, the results indicated that nitrogen source metabolism can regulate carbon source metabolism.

To summarize, this study found that the transcription factor GCN4 positively regulates nitrogen utilization in *G. lucidum* by directly binding to the promoter of the *GlMPC* gene and activating *GlMPC* expression. Furthermore, both GlMPC and GCN4 positively regulate the TCA cycle, thereby regulating secondary metabolism in *G. lucidum*. These findings provide a reference for studying the mechanism of MPC participation in nutrient utilization in other fungi.

## MATERIALS AND METHODS

### Experimental strains and culture conditions

*Escherichia coli* DH5α and Rosetta (DE3) for plasmid amplification and expression were grown in Luria-Bertani (LB) media containing 100 μg·mL^−1^ ampicillin or 50 µg·mL^−1^ kanamycin, as required. The *G. lucidum* strain was obtained from the Agricultural Culture Collection of China. The WT, Si-control (strain transformed with an empty vector), *GlMPC*-silenced (GlMPC1i-8, GlMPC1i-15, GlMPC2i-4, and GlMPC2i-5), *GCN4*-silenced (GCN4i-1 and GCN4i-22) strains have been constructed and described previously (3, 18) and were cultured in CYM (1% [wt/vol] maltose, 2% [wt/vol] glucose, 0.2% yeast extract, 0.2% tryptone, 0.05% MgSO_4_·7H_2_O, and 0.46% KH_2_PO_4_ with an initial pH 5.5) at 28°C. The silencing efficiencies of the GCN4i-1 and the GCN4i-22 strains were 79% and 81%, respectively. In addition, the silencing efficiencies of the *GlMPC1*-silenced strains and the *GlMPC2*-silenced strains were 70% and 65%, respectively. Based on previous studies, the nitrogen composition on the CYM medium was replaced with 3 mM or 60 mM asparagine (Asn) as the only nitrogen. Asn at 3 mM and 60 mM simulated nitrogen-limited and nutrient-replete conditions, respectively (3, 49).

### Quantitative PCR analysis

Total RNA was prepared from 100 mg hyphae using the RNAiso Plus Reagent (TaKaRa, Dalian, China), and the 5× All-In-One RT MasterMix kit was used for the cDNA synthesis (AccuRT Genomic DNA Removal Kit, ABM, Canada). Based on our previous studies (58), we used quantitative real-time PCR to detect gene-specific mRNA levels of WT and RNAi transformant strains on an Eppendorf Mastercycler ep Realplex 2.2 software (Eppendorf, Hamburg, Germany) using EvaGreen 2× qPCR MasterMix (ABM). Gene expression was evaluated by calculating the difference between the threshold (CT) value of the analyzed gene and that of the housekeeper gene 18S rRNA. The gene fragments were amplified by real-time PCR using primers as shown in Table S2.

### Extraction of mitochondria in *G. lucidum*

The extraction scheme of mitochondria in *G. lucidum* was based on previous reports (59, 60). The mitochondrial extraction process was conducted at 4°C. A quantity of fermented mycelium powder, ground using liquid nitrogen, was weighed, and mixed with triploid mitochondria extraction buffer. The mixture was then shaken and allowed to stand in an ice bath for 30 min. Following this, the mixture was centrifuged at 4°C and 1,200 *g*·min^−1^ for 15 min. The resulting supernatant was carefully transferred to another centrifuge tube, which was subsequently centrifuged at 4°C and 17,000 *g*·min^−1^ for 20 min. The resulting precipitates were washed twice using mitochondrial extraction buffer to obtain the purified mitochondria.

### Extraction and determination of pyruvate

The extraction scheme of pyruvate is based on previous reports (61). In this study, pyruvate was extracted from intracellular and mitochondrial sources at a temperature of 4°C. The extracted mitochondria or mycelium powder was suspended in 80% ethanol and subjected to ultrasonic treatment using an ultrasonic cell crusher at 4°C. The ultrasonic power was 20%, the ultrasonic was 10 s, the interval was 10 s, and the total ultrasonic was 10 min. Following ultrasonication, the sample was centrifuged at 4°C and 8,000 *g*·min^−1^ for 10 min, and the resulting supernatant was collected as the pyruvate solution. The pyruvate content was determined using ultraperformance liquid chromatography (UPLC), and the detection method followed the scheme described in previous reports (62).

### Measurement of GA content

The extraction and determination of total GA were performed by a previously described method (63). Mycelia were cultivated with a two-stage cultivation strategy, and the mycelia with different treatments were collected at the same time for GA extraction (3). The fermented mycelium of *G. lucidum* was dried at 60°C and ground into powder. Briefly, 0.2 g of samples was taken and ultrasonicated with 10 mL 95% (wt/vol) ethanol for 2 h, shaken every 20 min, and then centrifuged at 4,000 rpm·min^−1^ for 10 min. Then, 8 mL supernatant was dried at 60°C. Finally, 1 mL methanol was added to dissolve the residue. A UPLC system (Agilent Technologies, Santa Clara, CA, USA) was used to determine the GA content.

### Y1H

The JASPAR database (http://jaspar.genereg.net/) was used to analyze the putative transcription factors GCN4-binding sites. The Y1H assay was performed according to the protocol of the Matchmaker one-hybrid system (Clontech/Biosciences, Palo Alto, CA, USA). DNA fragments of *GlMPC* promoter regions that contain the GCN4-binding sites and promoter regions containing the mutated motif were cloned into the pAbAi vector (Invitrogen, Carlsbad, CA, USA), and the pGADT7-*GCN4* vector has been described previously (3). Plasmids were linearized by *Bst* BI (New England Biolabs) digestion and transformed into Y1HGold yeast strains to generate reporter strains (64). Strains were cultured in a synthetically defined medium lacking uracil (SD/-Ura) at 30°C for 3 days to screen transformants, and colony PCR was used to verify transformants. Then, the resistance concentration of aureobasidin A (AbA) was determined in transformants. Then, pGADT7-*GCN4* and empty negative-control vector were transformed into the above-described Y1HGold yeast transformants and plated on SD/-Leu plates. Plates were incubated at 30°C for 3 days. Transformants were plated onto SD/-Leu and SD/-Leu/AbA plates separately and cultured for 2–3 days at 30°C to detect the transcriptional activation effect. All primers used are listed in online supplementary file 1.

### ChIP

ChIP assays were performed as described previously (65, 66). Briefly, 1-week-old mycelia treated in 3 mM Asn or 60 mM Asn sample (2 g) were treated with 1% formaldehyde for protein-DNA cross-linking. The samples were ground and the chromatin was extracted with anti-GCN4 antibody. The protein A-agarose beads were used for purifying the DNA-histone-antibody complex. Finally, the enriched DNA fragments were analyzed by qPCR. All primers used are listed in the supplemental material.

### EMSA

For EMSA, the DNA fragments of GlMPC were amplified using primers labeled with 5′-biotin probes (supplemental file). Expression and purification of GCN4 proteins followed previous methods (3). The recombinant GCN4 proteins (0–8 μg mL^−1^) were incubated in binding buffers (10  mM Tris-HCl [pH 8.0], 1  mM dithiothreitol, 0.1  mM EDTA, 50  mM KCl, and 5% glycerol) with labeled DNA probes (10 ng) for 30 min at 25℃. A protein-free mixture was set as a negative control. For competition experiments, 100-fold diluted unlabeled DNA fragment probes are added to the reaction. The above-mixed samples were separated using nondenaturing 6% polyacrylamide gels with 0.5× Tris-borate-EDTA running buffer. The following steps were completed using the chemiluminescent nucleic acid detection module kit (Thermo Fisher Scientific, USA). Images were acquired using the Bio-Rad ChemiDoc Touch imaging system (3).

### Statistical analysis

Statistical analysis in this study was performed using GraphPad Prism 6. All experimental data in this paper were obtained from three separate samples to ensure that the trends and correlations observed in the culture are repeatable. Each error bar represents the mean of the standard deviation of the three replicates. Data were analyzed by multiple comparative one-way analysis of variance. Different letters correspond to *P* < 0.05.

## Data Availability

The data that support the findings of this study are available from the corresponding author upon reasonable request.

## SUPPLEMENTAL MATERIAL

Tables S1 and S2 mbio.01356-23-s0001.docx Screening and identification of GlMPC1-interacting proteins；primers used in the experiment.

ASM does not own the copyrights to Supplemental Material that may be linked to, or accessed through, an article. The authors have granted ASM a non-exclusive, world-wide license to publish the Supplemental Material files. Please contact the corresponding author directly for reuse.
