# Supplementary material for: GlMPC activated by GCN4 regulates secondary metabolism under nitrogen limitation conditions in Ganoderma lucidum
Source: mBio. 2023 Sep 21;14(5):e01356-23. doi: 10.1128/mbio.01356-23 (PMC10653791; doi:10.1128/mbio.01356-23)
Supplement: Tables S1 and S2 — Screening and identification of GlMPC1-interacting proteins；primers used in the experiment. [file mbio.01356-23-s0001.docx]

Supplementary materials for

*GlMPC* Activated by GCN4 Regulates Secondary Metabolism under Nitrogen Limitation Conditions in *Ganoderma lucidum*

Zi Wang#, Juhong Chen#, Juan Ding, Jing Han, Liang Shi*

Key Laboratory of Agricultural Environmental Microbiology, Ministry of Agriculture; Microbiology Department, College of Life Sciences, Nanjing Agricultural University, Nanjing, Jiangsu, 210095, China.

*For correspondence. E-mail: shiliang@njau.edu.cn; Tel./Fax 0086-25-84395602

#These authors contributed equally to this work. Author order was determined in order of increasing seniority.

**Table S1.** Screening and identification of GlMPC1-interacting proteins

| **Interacting proteins** | **Function descriptions** |
| --- | --- |
| EXS, C-terminal | Integral to membrane |
| COG5139 | Function unknown |
| Fungal_trans | DNA binding; Function unknown |
| WD40 | Function unknown |
| CDP-OH_P_transf | Phospholipid biosynthetic process; phosphotransferase activity, for other substituted phosphate groups |
| Glyceraldehyde 3-phosphate | Glyceraldehyde-3-phosphate dehydrogenase (phosphorylating) activity |
| Cytochrome P450 | Monooxygenase activity |
| COG5580 | Response to stress |
| RicinB_lectin_2 | Ricin-type beta-trefoil lectin domain-like |
| ENTH_Ent3 | Phosphoinositide binding site |
| HSP20 | Small heat-shock-proteins |
| bZIP_GCN4 | Transcription factor activity |
| DUF5648 | Function unknown |
| AhpC-TSA | Antioxidant activity, Molecular Function: oxidoreductase activity |
| bHLHzip_Myc | Transcription regulator activity |

**Table S2.** Primers used in the experiment

| **Primers** | **Primer sequences (5′ to 3′)** | **Description** |
| --- | --- | --- |
| RT-18S-F | TATCGAGTTCTGACTGGGTTGT | Detects the *18S* expression |
| RT-18S-R | ATCCGTTGCTGAAAGTTGTAT |  |
| RT-GlMPC1-F | TGTCACGCAACGAACGC | Detects the *MPC1* expression |
| RT-GlMPC1-R | TTGAGACGGAGACGGCC |  |
| RT-GlMPC2-F | TAGCTTGGCTGCTGTCA | Detects the *MPC2* expression |
| RT-GlMPC2-R | GCACCATCGGATTTCTG |  |
| RT-ICDH1-F | CGATTCGTGGGTTTGCC | Detects the putative *ICDH1* expression |
| RT-ICDH1-R | CGGTGCTCGTAATAGATGC |  |
| RT-ICDH2-F | TCAACACCGTCCTCATCC | Detects the putative *ICDH2* expression |
| RT-ICDH2-R | CTCAGAAGCGTCCCAAGT |  |
| RT-KGDH1-F | GCTCGCTGCTGACTGGC | Detects the putative *α-KGDH1* expression |
| RT-KGDH1-R | ATGCGTGGCTGCGTGAA |  |
| RT-KGDH2-F | CACGCAGGCGATGGAAA | Detects the putative *α-KGDH2* expression |
| RT-KGDH2-R | AGCGGGACGACGACACC |  |
| MPC1-F | CAGCCCCACCCTTGGAATGACTAATTTGCCGCAGAGTGGGA | GCN4 binding sequence in *GlMPC1* promoter fragment |
| MPC1-R | TCCCACTCTGCGGCAAATTAGTCATTCCAAGGGTGGGGCTG |  |
| MPC1-T-F | CAGCCCCACCATATATATATATATATATATACAGAGTGGGA | Mutant GCN4 binding sequence in *GlMPC1* promoter fragment |
| MPC1-T-R | TCCCACTCTGTATATATATATATATATATATGGTGGGGCTG |  |
| MPC2-F | GGAGATCGAGTTGGAGACGAGTCATTCACGATGACGAGAGC | GCN4 binding sequence in *GlMPC2* promoter fragment |
| MPC2-R | GCTCTCGTCATCGTGAATGACTCGTCTCCAACTCGATCTCC |  |
| MPC2-T-F | GGAGATCGAGTTGGAGACATATATTTCACGATGACGAGAGC | Mutant GCN4 binding sequence in *GlMPC1* promoter fragment |
| MPC2-T-R | GCTCTCGTCATCGTGAAATATATGTCTCCAACTCGATCTCC |  |
| MPC1-F | CTAGAAGCTTCACGTGAGGCTGG | Amplify the promoter fragment of *GlMPC1* |
| MPC1-R | TTAAGGTACCGACGGGAGTGG |  |
| ChIP-MPC1-F | GGCCGCTCCGCGGCCCA | Primers for ChIP-qPCR of *GlMPC1* |
| ChIP -MPC1-R | AGGCAGTCACCAGTCTG |  |
| ChIP -MPC2-F | CGCTGGACACATTGAGC | Primers for ChIP-qPCR of *GlMPC2* |
| ChIP -MPC2-R | AGGGCGCAAAGTCTTA |  |
| MPC1-F | CCAGCCCCACCCTTGGAATGACTAATTTGCCGCAGAGTGGGAC | Amplify GCN4 binding sequence in *GlMPC1* promoter fragment |
| MPC1-R | TCGAGTCCCACTCTGCGGCAAATTAGTCATTCCAAGGGTGGGGCTGGTAC |  |
| ***Continued*** | | |
| **Primers** | **Primer sequences (5′ to 3′)** | **Description** |
| MPC2-F | CGGAGATCGAGTTGGAGACGAGTCATTCACGATGACGAGAGCC | Amplify GCN4 binding sequence in *GlMPC2* promoter fragment |
| MPC2-R | TCGAGGCTCTCGTCATCGTGAATGACTCGTCTCCAACTCGATCTCCGGTAC |  |

**The promoter fragment of *GlMPC1***

CACGTGAGGCTGGCGAGGCAGCTCGAGAAGTGATTGCGCCTGCTTCGACGCTTGCGAGGACACGTTTAGAAGAGGAAATATATTATGGATAGTAGGCTAAGCACCTAGTGGTATAATTGAGGGAGGGGAGCCGGCAAGTCCAGGCCAATAACAATGTAAGTGTGACCTCAACTGCAACGCAAATCACGGGGGACCGAGGATGCCTGGAGGGGAGAACCTGGTCTCGTTCGAAAGGTGTGCTTCGCGCCTACAAAATCGGCCTAAAATCAACGAAAACTGTGGAGAACTGGGGGAGCAACTGTTGACGCAAACTGATGATGGGCGTCAAAATCAGAGCACCCGATGGCAAGCGTAATAGAGCACCATTGGAAGCTCTGAAAATTGAGAGAAATATATTCATAGAAAGTAAAGGAGCTACAAGTGATTGGGCAGAAAGAAGCGTAGAAATGCGATTATAATTGAAGGACATACGCAATTCGCACCAGGGGCCAATATTTGGAGTATATTTTCAAGCTCCAAAGCACCAGAACTTGTCTGATAGTAGTTCTGAGGTCCACATACATCAAAGCTAAATGCTATGCACAGATCGCGATATGCCAGGCTTCCAAACTCGAGGATCCGGAGTGGATGGAAGCCCGGAGGTGAGCCATCAAGCTGTAGACTGAATGTTGAGTGACAGAAGGAGACGCGGCAGCGCGCGCGCCGCCGCGAGAAGCGAAATATTTAGCAACATATTGTAGTCTTTTTTGCAGTACAGTCGGGGGAGAGCAGGGATGTTGGTCCGGTGGGGGGAGGGGAGCGCGCCTTCGGCGCGCGACCCCGCGCGAGCTCCCTGGGCAGCTTTTCAGCGCCTTGGGGCCGCTCCGCGGCCCAGCCCCACCCTTGGAATGACTAATTTGCCGCAGAGTGGGACCCACAGGCGCCGCACCGAGTCGGACGGTTGCACAAATGCTGCTCGTCAGACTGGTGACTGCCTTGCACTTGCACCCCCACTCCCGTC

**The promoter fragment of *GlMPC2***

CAGGGAAGCAGATGCTTGTTCGCGAGAGGAGCATCGGTAGCCTTCACGTTTGAGCCCTGATTGGCAGGTATAACTCACATAAAGGCACCAATCGAGCTCCGTGGTGCAAGACTGGCCAACTTGAAGCCGTAGTGATGAAATTCATTGGTCGAATAGATTTCGTCGCTGCATTGGTGGCGTTCGGGCAGGCATTCGACAGCAACGAGGCTCGCAAGAGGCTACGTGGCCGACCGTTTCATCTTTCATGGATGCTGTCAGTTTTGCTTCGTGCTCAGGACAAGCGTTCGGCATACCGCATGCTGGATTATGTCCGATGACCAACATTCGTCGCAGGCGTCCGTGGAGCTGGCCGCCCGTGGGTGCACGAAGGGGGCCTCGGTGTCCATCTGTCCATCGAAGGAGTGAAGACATGCCCTTCGACATTGAGAAGGAGCTAGGCAAAATCACGGGTAGCTCCAAAATTTCTACAGAATCAAAGCAACTCAGAGGAGCGTCACCTCAACTCGCGCAAGACCGTAGGACATACCGCTATCACATGTGTACCGTCTGAAGTTTGGCTGCCCATCCAGTTGAAGCGGTTTCAATTTGTTTTCGACTTCGACTCGGAAGTCTGAGCGTGCGCGTGAGCTGGGGCAAAGGTGCGCATATACTAAGTACTATATAATCATACCGACCGGGCTAGGTTCGAGTGGCCAAATATAGATCTTGGTAATACTTAAACGACGTTGTCCGTTTGCGCTGGACACATTGAGCTACTTATCCATGTCAGGAGATCGAGTTGGAGACGAGTCATTCACGATGACGAGAGCGTCCGACTACCATGGCGTGGGTAAGACTTTGCGCCCTCCTGACATCCATGCCGGCGCTAAGCCACCCCACCGCCAAATCGCCCCACACCCATCCCGTCGCCCTTGCGTCGCCCTCATCCGACCGGCCTTGCCCCGCCCTCCTTCCATCCACCACCACCCAGCCACCACCTAGACGTGCTCTAATCTCAATA
